# Expert Consensus Statement on Simplified Glycemic Care in Patients With Type 2 Diabetes Mellitus

**DOI:** 10.7759/cureus.87002

**Published:** 2025-06-29

**Authors:** Bipin Sethi, Subhankar Chowdhury, Sunil M Jain, Abdul Hamid Zargar, Manoj Chadha, Arpandev Bhattacharyya, Shehla Shaikh, Brij Mohan Makkar, Manoj Chawla, Pramila Kalra, Rishi Shukla, Sailesh Lodha, Sambit Das, Anuj Maheshwari, Surya K Singh, KM Suryanarayana, Jayashree Swain, Nitin R Gupta, Manoj Kumar Shrivastava, Nilakshi Deka, Dinesh Jiwane, Sanjay Jain, Onkar C Swami

**Affiliations:** 1 Endocrinology, CARE Hospitals, Hyderabad, IND; 2 Endocrinology, Medica Superspecialty Hospital, Kolkata, IND; 3 Endocrinology, Totall Diabetes Hormone Institute, Indore, IND; 4 Endocrinology, Centre for Diabetes and Endocrine Care, Srinagar, IND; 5 Endocrinology, P. D. Hinduja National Hospital and Medical Research Centre, Mumbai, IND; 6 Endocrinology, Manipal Hospital, Bengaluru, IND; 7 Endocrinology, Saifee Hospital, Mumbai, IND; 8 Endocrinology, Dr. Makkar’s Diabetes and Obesity Centre, New Delhi, IND; 9 Diabetes and Endocrinology, Lina Diabetes Care Centre, Mumbai, IND; 10 Endocrinology, Ramaiah Medical College and Hospital, Bengaluru, IND; 11 Endocrinology, Regency Hospital, Kanpur, IND; 12 Endocrinology, Eternal Hospital, Jaipur, IND; 13 Endocrinology, Kalinga Institute of Medical Sciences, Bhubaneswar, IND; 14 General Medicine, Hind Institute of Medical Sciences, Lucknow, IND; 15 Endocrinology, Institute of Medical Sciences, Banaras Hindu University, Varanasi, IND; 16 Endocrinology, Institute of Medical Sciences and SUM Hospital, Bhubaneswar, IND; 17 Endocrinology, Lucknow Hormone Centre, Lucknow, IND; 18 Internal Medicine, Kashi Medicare, Lucknow, IND; 19 Endocrinology, Apollo Hospital, Guwahati, IND; 20 Medical Services, Alembic Pharmaceuticals Ltd., Mumbai, IND

**Keywords:** combination therapy, dipeptidyl peptidase-4 (dpp-4) inhibitors, lifestyle modifications, monotherapy, sodium-glucose cotransporter-2 (sglt-2) inhibitors, type 2 diabetes mellitus (t2d)

## Abstract

The growing burden of type 2 diabetes mellitus (T2D) in India, characterized by rising prevalence, complex treatment regimens, and substantial economic and psychological impact, necessitates a simplified, patient-centered approach to glycemic management. This expert consensus document presents evidence-based recommendations for streamlined glycemic care focused on enhancing treatment adherence, minimizing pill burden, and improving clinical outcomes. An expert panel of endocrinologists and diabetologists convened across eight advisory board meetings to review current evidence and generate practical strategies. Emphasis was placed on the early use of combination therapies, particularly sodium-glucose cotransporter-2 (SGLT-2) inhibitors and dipeptidyl peptidase-4 (DPP-4) inhibitors, with or without metformin, tailored to individual patient profiles. The consensus underscores the importance of fixed-dose combinations (FDCs) to improve adherence and cost-effectiveness. In addition to pharmacologic strategies, lifestyle interventions, including medical nutrition therapy, physical activity, and sleep hygiene, are advocated. Special considerations are provided for managing T2D with comorbid conditions such as cardiovascular disease, chronic kidney disease, hypertension, obesity, and dyslipidemia, emphasizing the pleiotropic benefits of SGLT-2 inhibitors and glucagon-like peptide-1 (GLP-1) receptor agonists. The recommendations also address barriers in the Indian healthcare landscape, including limited access to care and treatment inertia. This consensus aims to support clinicians, researchers, and policymakers in implementing an integrative, simplified care model that addresses the multifaceted challenges of T2D management in India. By adopting these recommendations, healthcare providers can enhance patient outcomes, reduce complications, and alleviate the socioeconomic burden of T2D.

## Introduction and background

Diabetes presents a growing health crisis and is rapidly becoming a potential epidemic in low- and middle-income countries (474.7 million), including India. Globally, approximately 588.7 million people are currently living with diabetes, and this number is projected to rise to 852.5 million by 2050 [1]. According to the Indian Council of Medical Research-India Diabetes-17 (ICMR-INDIAB-17) study, 101.3 million individuals in India are currently living with diabetes. The overall prevalence of diabetes in India is 21.1% (11.4% by oral glucose tolerance test (OGTT) and 13.3% through glycated hemoglobin (HbA1c) testing) [2]. The National Family Health Survey (NFHS-5) reports a diabetes prevalence of 16.1% (15.9-16.1%) in India [3]. The International Diabetes Federation (IDF) estimates that 11.1% of adults aged 20-79 have diabetes [1]. Meanwhile, the Longitudinal Aging Study in India indicates that 14% of the elderly population is living with type 2 diabetes mellitus (T2D) [4].

T2D presents a significant health burden in India, further exacerbated by its association with serious complications (macro- and microvascular). These complications worsen patient outcomes and place a substantial financial burden on individuals, families, communities, and the overall healthcare system [5]. A systematic review by Oberoi and Kansra [6] (32 studies) found the mean direct annual cost of managing diabetes in India to range from Rs 3,949 to 45,792, reflecting the economic strain T2D places on households. In addition to financial pressures, the daily management of T2D requires significant restraint and discipline, which can lead to mental fatigue, stress, burnout, and even depression [7]. A systematic review and meta-analysis conducted by Sinha et al. [8] (10 studies, n=2,100) reported that diabetic distress affects 33% (95% CI: 21%-45%) of people with T2D in India. Newer oral antihyperglycemic agents (OHAs) in combination therapies with or without metformin (considered first-line by various organizations) [9-11] can help reduce the pill burden, improve adherence, and lower costs while providing additional cardiovascular and renal protection [12,13].

Despite the availability of various OHAs, a large proportion of individuals with T2D in India fail to achieve optimal glycemic control. Studies have identified multifactorial determinants, including treatment inertia, inadequate physician engagement, and poor medication adherence due to pill burden, side effects, and affordability. Psychosocial stressors, such as diabetes distress and depression, are prevalent and often go unrecognized. Lifestyle-related contributors, including urbanization, unhealthy dietary habits, and physical inactivity, further compound the problem. The shortage of trained healthcare professionals, especially certified diabetes educators, poses a significant barrier to effective, sustained glycemic control. In rural and underserved regions, these workforce gaps are more pronounced, resulting in limited patient counseling, inadequate follow-up, and poor treatment adherence. Together, these challenges underscore the need for a simplified, scalable care model tailored to India’s diverse healthcare landscape [14].

These challenges highlight the need for a simplified approach to glycemic care, focusing on a streamlined, patient-centered method of managing blood glucose levels. This approach seeks to reduce the complexity of treatment regimens, enhance patient adherence, and ultimately improve clinical outcomes. The need for such an approach is particularly pressing in India, where the prevalence of T2D is high, and many patients face barriers to effective care, including limited access to healthcare in rural areas, economic constraints, a lack of awareness and education regarding diabetes management, and complex treatment protocols.

This consensus document aims to establish a framework for simplified glycemic care in India, focusing on sodium-glucose cotransporter-2 (SGLT-2) inhibitors, dipeptidyl-peptidase-4 (DPP-4) inhibitors, and their combinations. It provides clinical recommendations developed by a distinguished group of endocrinologists, diabetologists, and physicians to enhance patient adherence, optimize clinical outcomes, and alleviate the economic and psychological burdens associated with T2D in the Indian context.

## Review

Materials and methods

This consensus involved a group of healthcare professionals (HCPs) developing strategies for simplified glycemic care in individuals with T2D. The objective was to gather expert opinions to create strategies that focus on combination therapy, reduce pill burden, and enhance adherence to antihyperglycemic treatment. An expert committee of 10 key opinion leaders was formed to moderate the advisory board meetings, selected for their extensive experience, significant research contributions, and active engagement in the management of T2D.

A total of eight advisory board meetings were conducted across India, featuring the 10 expert committee members as moderators and 85 panelists. Discussions focused on key topics, including newer dual and triple combination therapies and strategies for managing complications in T2D. To ensure the credibility and robustness of the evidence base, we primarily included randomized controlled trials (RCTs) and systematic reviews or meta-analyses published in peer-reviewed journals between 2019 and 2024. Meta-analyses were selected based on clearly defined research objectives, appropriate inclusion criteria, and adequate sample sizes, typically including more than five studies (mainly RCTs). We prioritized those that reported key clinical outcomes such as HbA1c, fasting plasma glucose, body weight, and cardiovascular endpoints. Additionally, statistical reliability was considered, with preference given to studies demonstrating low variability between studies and those following established reporting standards like Preferred Reporting Items for Systematic Reviews and Meta-Analyses (PRISMA). These criteria helped us ensure that the included studies were methodologically sound and relevant. Keywords such as “T2DM,” "Type 2 Diabetes Mellitus," “SGLT2i,” "Sodium-glucose cotransporter-2 inhibitors," “DPP-4 inhibitors,” “Biguanides,” “Metformin,” and “GLP-1 RA” were utilized to identify recent and relevant findings. The search focused on identifying best practices for improving treatment adherence, simplifying regimens, and addressing the economic challenges faced by patients.

The insights from the literature review and advisory board meetings were consolidated into a comprehensive document that outlined current knowledge and identified gaps in T2D management. This document was reviewed by the expert committee, and their feedback was incorporated into a final manuscript. The finalized consensus document provides actionable, evidence-based recommendations to support clinicians, researchers, and policymakers in enhancing treatment outcomes for individuals with T2D.

Management of type 2 diabetes mellitus

Individuals with T2D should be educated on the importance of regular glucose monitoring, medication adherence, and maintaining scheduled clinic visits for optimal management. They need guidance on making healthy dietary choices, staying physically active, and recognizing symptoms of high or low blood sugar. Effective diabetes management involves a combination of behavioral and pharmacological treatments to prevent or delay complications while preserving quality of life. This includes patient education, behavior interventions, medical nutrition therapy (MNT), physical activity, and pharmacologic management of blood glucose levels, weight, cardiovascular risk factors, comorbidities, and complications (Table 1) [11]. Table 2 details the consensus recommendations.

**Table 1:** Non-pharmacologic management strategies based on national and international guidelines and scientific evidence. MUFA: monounsaturated fatty acids; PUFA: polyunsaturated fatty acids; FBG: fasting blood glucose; AHEAD: Action for Health in Diabetes; WMD: weighted mean difference; HbA1c: glycated hemoglobin; MNT: medical nutrition therapy

Management strategy	Characteristics	Evidence
Medical nutrition therapy	Carbohydrates: 50-60% (low glycemic index and glycemic load) - cereals, mixed grains, non-starchy vegetables, and whole pulses, including soybeans and salads	A systematic review and meta-analysis by Razaz et al. [15] (11 studies, n=1227), reported a significant reduction in FBG (WMD=-8.85mg/dl, 95% CI: -14.41, -3.28), HbA1c (WMD: -0.43%, 95% CI: -0.69, -0.17), weight (WMD: -1.54kg, 95% CI: -2.44, -0.64), cholesterol, etc. by MNT.
	Proteins: 15%-20% - low-fat dairy, eggs, fish, lean meats, and plant-based sources (pulses and lentils) [9]	
	Fats: <30% (saturated fats: <10%, MUFA: 10% energy + any calories left from carbohydrate portion; PUFA- 10 % of energy) [9,16,17]	A systematic review and meta-analysis by Dudzik et al. [18] (13 studies) reported that MNT improved HbA1c (−0.30% (−0.49, −0.12)) and FBG (−4.97 mg/dL (−6.24, −3.71)).
	Micronutrients: chromium, alpha-lipoic acid, magnesium, and zinc	
	Nutraceuticals [19]	A study by Guilbert et al. [20] has shown that MNT reduced FBG (-13.63 mg/dL) and HbA1c (-6.3%).
	Avoidance of alcohol and tobacco	
	Include fresh fruits and fiber-rich foods and limit processed foods, refined carbohydrates, non-nutritive artificial sweeteners, refined sugars, and trans-fat [21,22]	
	Eating patterns should be modified, and late-night dinners and munching should be avoided	
Physical activity	150 minutes/week of physical activity should be introduced gradually	Systematic reviews and meta-analyses have shown that aerobic exercise reduces HbA1c by 0.5-0.7% [23-25].
	≥ 30 minutes of moderate-intensity activity (swimming, cycling, walking)	
	15-30 minutes of work-related	Combined aerobic and resistance exercise training reduced HbA1c (MD -0.17%) [26].
	15 minutes: muscle strengthening exercise (three times/ week)	
	5000 steps per day	Dietary restriction and increased physical activity improved HbA1c and cholesterol levels, according to the Look AHEAD trial [27].
	Yoga can be practiced [9]	
Sleep	Seven to eight hours of sleep is recommended	According to a meta-analysis (10 articles), individuals who slept at least seven to eight hours daily had a lower risk of diabetes. Both long and short durations of sleep impacted blood glucose levels [28].
	Sleeping: one to two hours after dinner.	Azharuddin et al. [29] and Lee et al. reported that short and long sleep durations and poor sleep quality are associated with higher HbA1c and FBG levels [30].
	Avoid using mobile phones, coffee, tea, and alcohol before going to sleep.	

**Table 2:** Consensus recommendations for diet and lifestyle modifications. HbA1c: glycated hemoglobin

Consensus recommendations
Individuals with prediabetes and also with HbA1c > 6.5 start with diet and lifestyle modifications.
Individualized nutrition plans should be developed.
Dietary modifications should include increased consumption of fiber, whole grains, lean proteins, and healthy fats while limiting refined sugars and saturated fats.
Individuals should aim for at least 150 minutes of moderate-intensity aerobic exercise per week, such as brisk walking, swimming, or cycling.
Encourage patients to maintain a regular sleep schedule.

Pharmacological management of type 2 diabetes mellitus

Guidelines recommend a comprehensive, individualized approach to managing T2D and its complications. Treatment decisions should consider medication tolerability, side effect profiles, the complexity of the treatment regimen, comorbid conditions, and factors such as access, cost, and availability of medications.

Monotherapy With Metformin

Metformin, a biguanide, is widely recommended as the first-line treatment for T2D due to its proven efficacy, affordability, and broad availability. This recommendation is endorsed by leading health organizations, such as the American Diabetes Association (ADA) [10], the European Association for the Study of Diabetes (EASD) [11], and the Research Society for the Study of Diabetes in India (RSSDI) [9]. Though studies have shown that metformin improves glycemic control, gastrointestinal side effects often limit its use. These include diarrhea, nausea, flatulence, indigestion, vomiting, and abdominal discomfort, with diarrhea and nausea being the most common issues [31].

Recommendations by guidelines: Along with lifestyle recommendations, metformin is the first-line drug, especially in individuals with obesity [9]. Metformin is considered for individuals at high risk of T2D, particularly those with a BMI ≥ 35 kg/m², individuals under 60 years of age, and women with a history of gestational diabetes [32]. If the estimated glomerular filtration rate (eGFR) is between 45 and 30 mL/min/1.73 m², reduce the dose by 50%, or avoid it if not on metformin. Discontinue metformin if eGFR is less than 30 mL/min/1.73 m² [9].

Evidence for metformin monotherapy: A systematic review and meta-analysis by Patel et al. [33] reported that metformin reduced the risk of developing diabetes in high-risk individuals. Individuals with prediabetes had 35% lower odds of developing T2D. Han et al. [34] and Hu et al. [35] reported that metformin significantly reduced the risk of all-cause mortality and cardiovascular events. The UK Prospective Diabetes Study (UKPDS) reported that overweight T2D individuals on metformin had less risk of all-cause mortality and diabetes-related deaths [36].

There is ongoing debate about whether metformin should continue to be the first-line therapy for all patients, as other antihyperglycemic medications have demonstrated additional benefits in certain populations. Understanding the risks and benefits of both metformin and alternative treatments is crucial before considering any changes to clinical practice [37,38]. In India, as fixed-dose combinations (FDCs) that commonly include metformin become increasingly popular, there is a growing need to explore effective and well-tolerated alternatives to metformin. Table 3 summarizes the consensus recommendations on monotherapy with metformin. Metformin should be prescribed if an individual's glucose levels do not improve with diet and lifestyle modifications and in individuals with HbA1c > 6.5%.

**Table 3:** Consensus recommendations for metformin monotherapy. HbA1c: glycated hemoglobin

Consensus recommendations
Metformin should be prescribed if an individual's glucose levels do not improve with diet and lifestyle modifications and in individuals with HbA1c > 6.5%.
Metformin is recommended for its proven cardiovascular benefits, including a reduction in the risk of myocardial infarction and overall cardiovascular mortality.
Adverse effects of metformin are rare and easily manageable.

Combination Therapy

**Figure 1:**
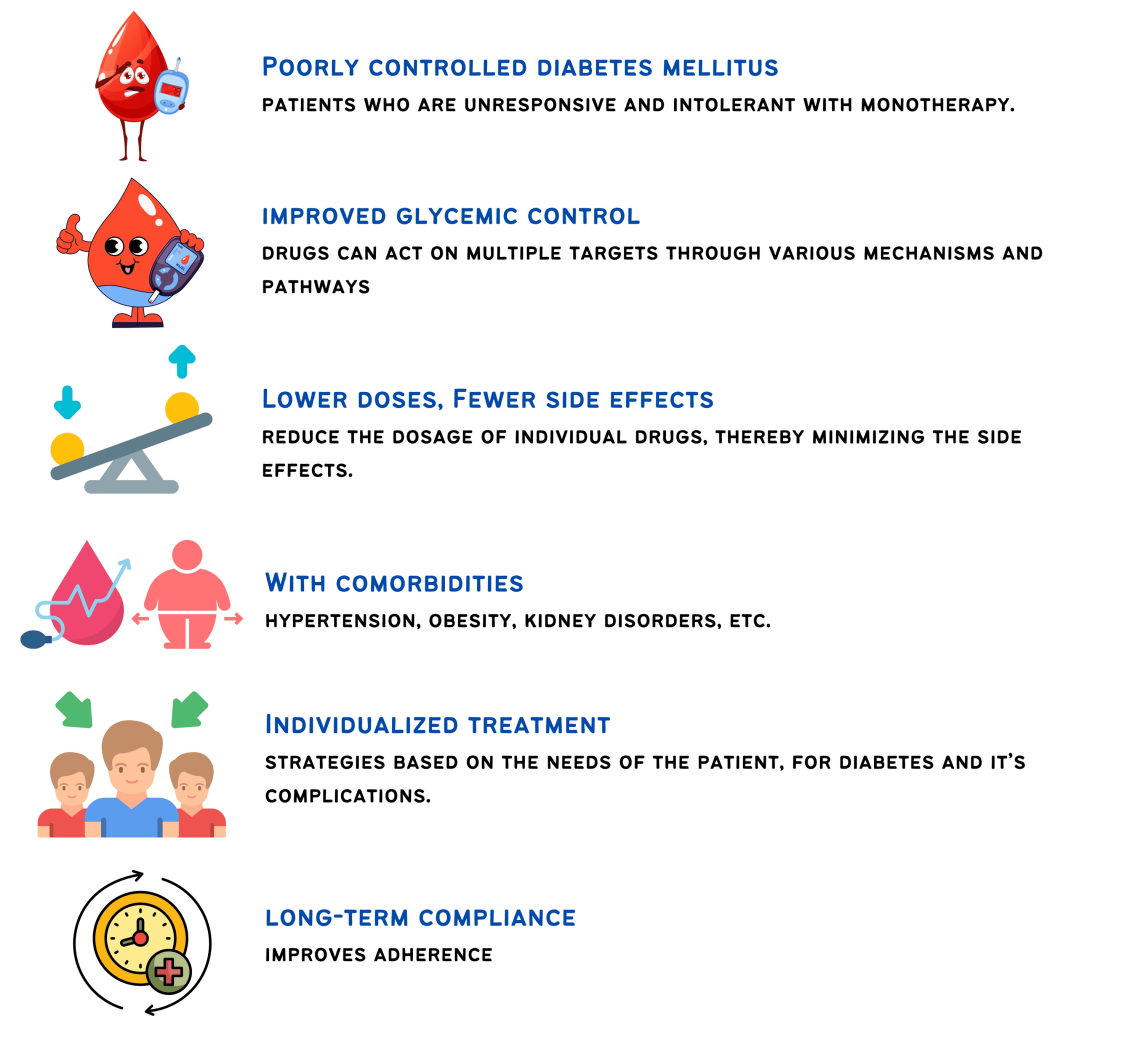
Indications of combination therapy for glycemic control. Image credit: Dr. Sanjay Jain

Monotherapy may be insufficient for achieving blood glucose control or preventing complications in individuals with diabetes due to the condition's complex pathogenesis. For individuals newly diagnosed with T2D who are drug-naive, research indicates that starting with combination therapy helps achieve glycemic targets more rapidly [39,40]. Early combination therapy is typically recommended for those with T2D and an HbA1c level of 7.5% or higher, often involving metformin and either a glucagon-like peptide-1 (GLP-1) receptor agonist, SGLT-2 inhibitor, or DPP-4 inhibitor. If HbA1c exceeds 9% or is more than 1.5% above the target, initiating more than two antihyperglycemic agents may be necessary (Figure 1) [41]. 

Recommendations by guidelines: The American Association of Clinical Endocrinology (AACE) recommends initiating combination therapy for patients with an HbA1c level above 7.5% [42]. ADA [10] suggests starting combination therapy when HbA1c is 1.5-2% higher than the patient’s glycemic target, while RSSDI [9] suggests HbA1c > 1.5 above target. If HbA1c levels remain above 7.5%, dual therapy is advised, and if there is no improvement within three months, triple therapy is recommended [42]. For individuals with an initial HbA1c level greater than 10% (> 300 mg/dL), injectable therapy should be considered [42]. A patient-centric approach should be considered when initiating combination therapy [9]. If target HbA1c levels are not achieved with three oral medications, adding a fourth agent with a complementary mechanism of action may be considered [9]. Table 4 details the consensus recommendations.

**Table 4:** Consensus recommendations for combination therapy. SGLT-2: sodium-glucose cotransporter-2; DPP-4: dipeptidyl peptidase-4; HbA1c: glycated hemoglobin

Consensus recommendations
Depending upon age, comorbidities, organ damage, and individualized, tailored therapy should be given.
Glucose control should be the primary focus, followed by regular screening and management of other comorbidities during follow-up visits.
Dual combination therapy is advised for individuals with an HbA1c level of > 8 % or when monotherapy does not achieve glycemic targets, or in patients who are intolerant to metformin.
Dual combination therapy of SGLT-2 inhibitors and DPP-4 inhibitors can be initiated as first-line therapy in individuals who are intolerant to metformin, and also depending on the individual's patient characteristics and comorbidities (cardiovascular and renal).
Triple therapy should be initiated if dual therapy fails to achieve target HbA1c levels within three months.
Dapagliflozin (10 mg) + sitagliptin (100 mg) + metformin (1000 mg) can be prescribed in individuals who do not achieve glycemic control with dual therapy and also in individuals with cardiovascular and renal risk factors.
Simplify the treatment regimen by considering fixed-dose combinations (FDCs) to reduce the pill burden and improve adherence.

Table 5 details evidence for combination therapy.

**Table 5:** Combination therapy for type 2 diabetes. SGLT-2: sodium-glucose cotransporter-2; DPP-4: dipeptidyl peptidase 4; MD: mean difference; HbA1c: glycated hemoglobin; FBG: fasting blood glucose; FPG: fasting plasma glucose; PPBG: postprandial blood glucose; INSITES: ProspectIve, OpeN-Label, Randomized Study Comparing EffIcacy and Safety of Teneligliptin VErsus Sitagliptin; DELIVER: Dapagliflozin Evaluation to Improve the LIVEs of Patients With PReserved Ejection Fraction Heart Failure

Dual combination therapy	
Combination	Evidence
Biguanide + DPP-4 inhibitors	A systematic review and meta-analysis by Chen et al. [43] (62 studies) reported that the combination therapy of DPP-4 inhibitors and metformin reported a greater mean reduction in HbA1c than other drug combinations. DPP-4 inhibitors and metformin combination reported lower incidence of cardiovascular events compared to sulphonylureas and metformin combination (RR: 1.06, 95% CI: 0.61, 2.06).
	A systematic review and meta-analysis by Janani et al. [44] (18 studies, n=2009), reported that a combination of sitagliptin and metformin reduced weight (MD -1.09; 95% CI; (-1.69, -0.49); p<0.001)) and BMI (MD -0.52; 95% CI; (-0.96, 0.08); p=0.020)).
	According to a meta-analysis by Ding et al. [45] (11 studies, n=8533), vildagliptin and metformin combination therapy reported a reduction in HbA1c (MD=-0.59, 95% CI (-0.28, -0.16), p<0.00001), body weight (MD=0.22, 95% CI (0.17, 0.27), p<0.00001).
	According to the INSITES study [46], individuals on teneligliptin/sitagliptin with background metformin therapy reported a reduction in HbA1c (-1.19 ± 1.16%), in FBG (-28.3 ± 63.0 mg/dL) and postprandial blood glucose (PPBG) (-41.3 ± 85.4 mg/dL).
	According to the EVOLUTION INDIA study [47], individuals on sitagliptin (100 mg) and metformin (>1000 mg) reported a reduction in HbA1c (-0.32 (1.14)) at the end of 12 weeks.
Biguanide + SGLT-2 inhibitors	A systematic review and meta-analysis by Neuen et al. [48] (six studies, n=51743), reported that SGLT-2 inhibitors with/without metformin reduced major adverse cardiovascular events (MACE) (HR 0.93, 95% CI 0.87–1.00), and hospitalization for heart failure (HHF) or cardiovascular death (HR 0.79, 95% CI 0.73–0.86).
	A systematic review and meta-analysis by Gebrie et al. [49] (nine studies, n=10974) reported that SGLT-2 inhibitor and metformin combination therapy reduced HbA1c (MD=−0.10%, 95% CI (−0.17, −0.03)), body weight (MD=−4.57 kg, 95% CI (−4.74, −4.39)), systolic blood pressure (MD=−4.77 mmHg, 95% CI (−5.39, −4.16)), diastolic blood pressure (MD=−2.07 mmHg, 95% CI (−2.74, −1.40)), and fasting plasma glucose (MD=−0.55 mmol/L, 95% CI (− 0.69, − 0.41)), p < 0.001.
	According to a systematic review by Scheen [50], SGLT-2 inhibitors and metformin combination therapy showed reduction in HbA1c (-0.60 and -0.54), FBG (-1.37 and -1.37), body weight (-2.23 and -2.16) in Asian vs. non-Asian individuals with T2D.
	Cuatrecasas et al. [51] reported that combination therapy of dapagliflozin and metformin decreased weight (-8.4 ± 4.4 kg), abdominal circumference (-5.4 ± 2.5 cm), and perirenal fat layer reduction.
	According to the prespecified analysis of the DELIVER trial [52], dapagliflozin in concomitant use of metformin improved clinical outcomes in individuals with T2D and heart failure with left ventricular ejection fraction (LVEF)> 40% (HR 0.73, 95% CI 0.59-0.92).
SGLT-2 inhibitors + DPP-4 inhibitors	A systematic review and meta-analysis by Li et al. [53] (14 studies, n=4828) reported that SGLT-2 inhibitor/ DPP-4 inhibitor combination therapy decreased HbA1c (−0.31%), and FBG (−8.694 mg/dL).
	An RCT by Jain et al. [54] reported a reduction in HbA1c (-1.28%) with the use of dapagliflozin and linagliptin.
	According to the DELIGHT study, individuals with T2D and chronic kidney disease on dapagliflozin (10 mg) and saxagliptin (2.5 mg) reported a reduction in HbA1c (-0.58% (-0.80 to -0.37; p<0.0001)) [55].
	Patients on a combination therapy of remogliflozin (100 mg) + vildagliptin (50 mg) and empagliflozin 25 mg + linagliptin 5 mg, reported a reduction in HbA1c (-1.46% and -1.38% respectively) at the end of 16 weeks [56].
Triple combination therapy	
Biguanide + SGLT-2 inhibitors + DPP-4 inhibitors	Singh et al. [57] reported that at 16 weeks, a fixed-dose combination of metformin (1000 mg), sitagliptin (100 mg), and dapagliflozin (10 mg), reduced HbA1c (-1.45%), FPG (∆-12.4 mg/dl; p=0.003), and PPBG (∆-18.45 mg/dl; p=0.01).
	Sahay et al. [58] reported that after 16 weeks, a fixed-dose combination of metformin (1000 mg), sitagliptin (100 mg), and dapagliflozin (10 mg) led to a reduction in HbA1c by 1.73%. Additionally, 38.5% of individuals using this combination achieved an HbA1c level below 7% by the end of the 16-week period.

Management of type 2 diabetes mellitus with co-morbidities

**Figure 2:**
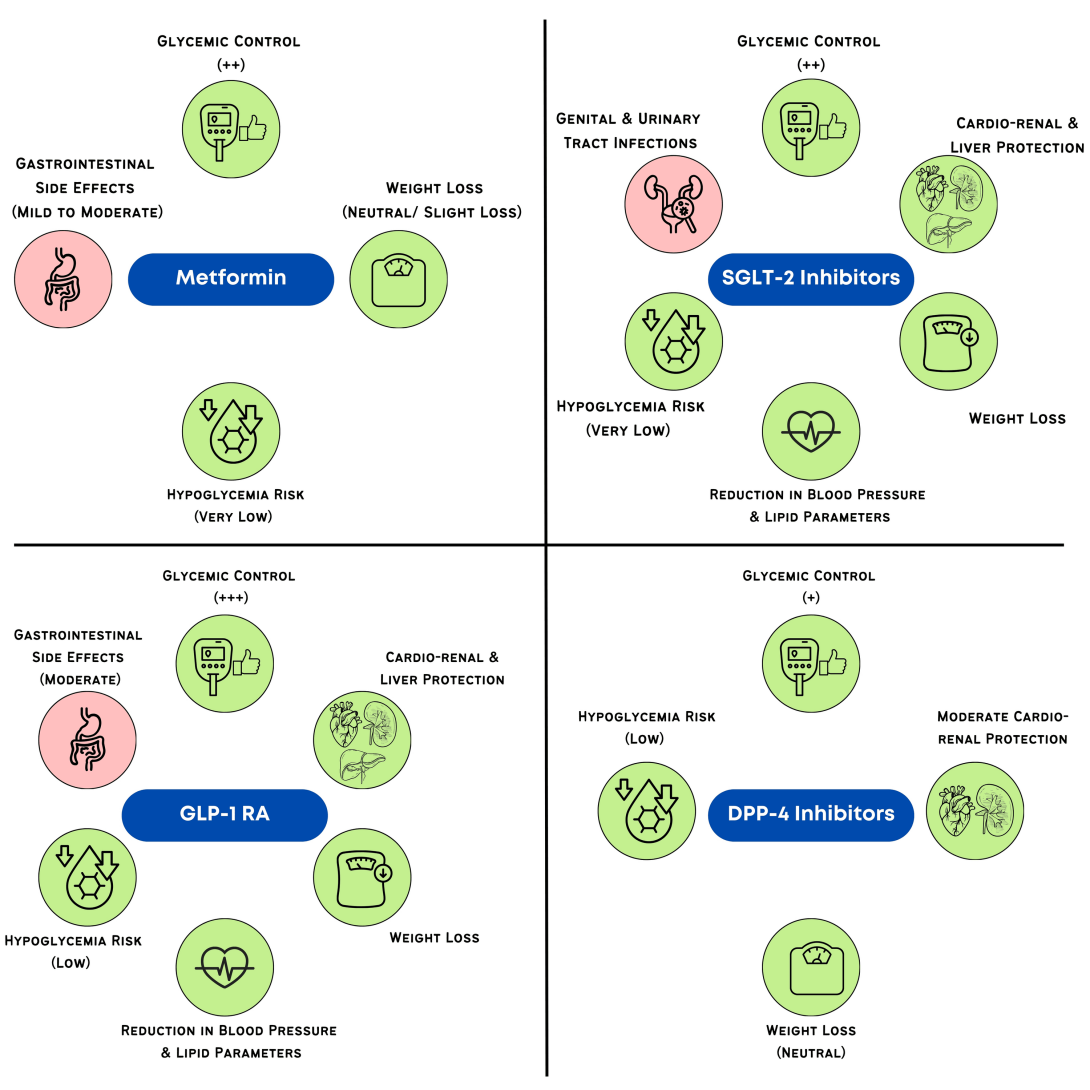
Benefits and adverse effects of common antihyperglycemic drugs for glycemic control. SGLT-2: sodium-glucose cotransporter-2; DPP-4: dipeptidyl peptidase 4; GLP-1: glucagon-like peptide-1 Image credit: Dr. Sanjay Jain

T2D often coexists with other chronic conditions such as cardiovascular disease (CVD), chronic kidney disease (CKD), hypertension, obesity, etc. Managing T2D in the presence of these comorbidities requires a comprehensive approach, as the interplay between these conditions can significantly influence treatment outcomes. An individualized treatment plan incorporating lifestyle modification, glycemic control, cardiovascular risk mitigation, and appropriate pharmacotherapy is essential. Various antihyperglycemic drugs offer benefits beyond blood glucose control, helping to mitigate comorbidities through their extraglycemic effects (Figure 2).

Type 2 Diabetes Mellitus With Hypertension

The combined management of T2D and hypertension is a clinical priority, as it can significantly reduce the risk of major cardiovascular events and microvascular complications. According to the National Family Health Survey (NFHS) data, the prevalence of hypertension in individuals with diabetes was 37% [59]. Managing individuals with T2D and hypertension requires a combination of increasing physical activity, modifying dietary habits, and using antihypertensive and oral antihyperglycemic medications. The ADA recommends that patients with both diabetes and hypertension who are at high cardiovascular risk may have their blood pressure lowered to < 130/80 mmHg, provided the target can be safely achieved. For blood pressure control, antihypertensive therapy with angiotensin-converting enzyme inhibitors (ACEIs) or angiotensin receptor blockers (ARBs) is advised [9,60].

Evidence for SGLT-2 inhibitors: A meta-analysis by Zhang et al. [61] (10 studies, n=9913) reported that SGLT-2 inhibitors reduced 24-hour systolic blood pressure (SBP) (-5.06 mmHg, p < 0.05), 24-hour diastolic blood pressure (DBP) (-2.39 mmHg, p=0.004), office SBP (-4.53 mmHg, p < 0.05), and office DBP (-2.12 mmHg, p=0.001). A meta-analysis by Iqbal et al. [62] (10 studies) reported that SGLT-2 inhibitors reduced 24-hour ambulatory SBP (WMD: -5.08 mmHg, 95% CI (-7.02 to -3.14), p < 0.00001)) and DBP (WMD: -2.73 mmHg, 95% CI (-4.25 to -1.20), p=0.0005). According to the Dapagliflozin Effect on Cardiovascular Events-Thrombolysis in Myocardial Infarction 58 (DECLARE-TIMI 58) trial, dapagliflozin reduced SBP (2.4 mm Hg) when compared to placebo at 48 months [63]. According to the Dapagliflozin on Left Ventricular Hypertrophy (DAPA-LVH) trial, dapagliflozin at the end of 12 months reduced ambulatory 24-hour SBP (-2.78±5.94 mmHg, p=0.012) and nocturnal SBP (-3.47±7.54 mmHg, p=0.017) [64]. An RCT conducted by Cheng et al. [65] reported that empagliflozin (25 mg) at the end of 12 weeks reduced 24-hour SBP (-8.14 mmHg (95% CI -10.32, -3.96, p=0.005)) and DBP (-5.27 mmHg (95% CI -8.19, -1.35, p < 0.001)).

Evidence for GLP-1 receptor agonists: A systematic review and meta-analysis by Kennedy et al. [66] (six studies, n=4744) reported that semaglutide reduced SBP (−4.83 mmHg, 95% CI (−5.65 to −4.02)), and DBP (−2.45 mmHg, 95% CI (−3.65 to −1.24)). A meta-analysis by Rivera et al. [67] reported that GLP-1 receptor agonists moderately reduced SBP (semaglutide (-3.40), liraglutide (-2.61), exenatide (-3.36)).

Evidence for combination therapy: A systematic review and meta-analysis by Mantsiou et al. [68] (seven studies, n=1913) reported that a combination of SGLT-2 inhibitors and GLP-1 receptor agonists reduced SBP (-4.13 mmHg (-7.28 to -0.99 mmHg)) when compared to GLP-1 receptor agonist monotherapy. Combination therapy also reduced SBP (-2.66 mmHg, (-5.26 to -0.06 mmHg)) compared to SGLT-2 inhibitor monotherapy. Table 6 details the consensus recommendations. 

**Table 6:** Consensus recommendations on management of T2D with hypertension. T2D: type 2 diabetes

Consensus recommendations
Diet and lifestyle modifications are to be made.
Use ACE inhibitors (ACEIs) or angiotensin receptor blockers (ARBs) as the first-line antihypertensive treatment in T2D patients with hypertension.
Consider sodium-glucose cotransporter-2 (SGLT-2) inhibitors along with metformin for glycemic and hypertension control.

Type 2 Diabetes Mellitus and Cardiovascular Disease

The major CVDs associated with T2D include ischemic heart disease, heart failure, stroke, coronary artery disease, and peripheral artery disease. These complications are responsible for mortality in at least 50% of patients with T2D [69]. Globally, the weighted prevalence of CVD among individuals with T2D stands at 34.8% [70]. In India, based on the QRESEARCH risk estimator version 3 (QRISK3) calculation, the average CVD risk for individuals with diabetes is 15.3 ± 12.3%, with males showing a significantly higher risk (17.1 ± 13.5%) compared to females (12.2 ± 10.1%) [71]. A comprehensive, multifactorial approach is essential to mitigate cardiovascular risk in patients with T2D. This strategy should include lipid management, glycemic control, blood pressure regulation, smoking cessation, weight management, and increased physical activity. GLP-1 receptor agonists and SGLT-2 inhibitors have been demonstrated to reduce atherosclerotic CVD (ASCVD) risk in persons with T2D [10,11].

Guidelines: Early use of SGLT-2 inhibitors and GLP-1 receptor agonists in managing high-risk T2DM patients may offer potential cardiovascular benefits [9]. According to the European Society of Cardiology (ESC) 2023 guidelines, SGLT-2 inhibitors and/or GLP-1 receptor agonists are recommended for individuals with T2D and cardiovascular disorders [72]. According to the European Society of Cardiology and the European Association for the Study of Diabetes (ESC-EASD) guidelines, among the available DPP-4 inhibitors, linagliptin and sitagliptin have neutral effects on the risk of hospitalization for heart failure (HHF) and may be considered for treating T2D individuals with heart failure [73]. For individuals with T2D and ASCVD or kidney disease, monotherapy with an SGLT-2 inhibitor or GLP-1 receptor agonist, or combination therapy with proven cardiovascular benefits, is recommended. This approach has demonstrated reductions in the risk of major adverse cardiovascular and kidney events, worsening heart failure, HHF, and cardiovascular death [60].

Evidence for SGLT-2 inhibitors: Usman et al. [74] conducted a systematic review and meta-analysis (15 studies, n=100952) and reported that SGLT-2 inhibitors reduced HHF by 28% in individuals with T2D and 28% in individuals with ASCVD. They reduced cardiovascular death by 14% in patients with heart failure (HR 0.86 (95% CI 0.79-0.93)), 15% in patients with T2D (0.85 (0.79-0.91)), and 13% in individuals with ASCVD (0.87 (0.78-0.97)). A systematic review and meta-analysis by Marilly et al. [75] (five studies, n=46,969) reported that SGLT-2 inhibitors decreased the risk of all-cause death (incidence rate ratio (IRR) 0.86 (95% CI 0.78, 0.95)), major adverse cardiovascular events (MACE) (IRR 0.91 (95% CI 0.86, 0.96)), and HHF (IRR 0.69 (95% CI 0.62, 0.76)) in individuals with T2D. According to Aziri et al. [76] (12 studies, n=83878), SGLT-2 inhibitors improved the quality of life in heart failure individuals (atrial fibrillation (OR=0.83, 95% CI: 0.68-1.01), HHF (OR=0.69, 95% CI: 0.60-0.78), cardiovascular death (OR=0.82, 95% CI: 0.58-1.15), and MACE (OR=0.90, 95% CI: 0.77-1.06)).

Evidence for GLP-1 receptor agonists: A meta-analysis by Rivera et al. [77] (13 studies, n=83258) reported that GLP-1 receptor agonists reduced MACE (OR 0.86, 95% CI (0.80 to 0.94), p < 0.01), all-cause mortality (OR 0.87, 95% CI (0.82 to 0.93), p < 0.001), CV mortality (OR 0.87, 95% CI (0.81 to 0.94), p < 0.001), and stroke (fatal and non-fatal). Villaschi et al. [78] (10 studies, n=68653) reported that GLP-1 receptor agonists reduced HHF (HR=1.00, 95% CI (0.82-1.24), p=0.12) and CV death (HR=0.97, 95% CI (0.81-1.15), p=0.11). and MACE (HR=0.83, 95% CI (0.72-0.95), p=0.69). In a meta-analysis conducted by Giugliano et al. [79] (eight studies, n=60080), GLP-1 receptor agonists reduced MACE (14%), cardiovascular death (13%), nonfatal stroke (16%), HHF (10%), and all-cause mortality (12%).

Evidence for DPP-4 inhibitors: A systematic review and meta-analysis by Liu et al. [80] reported that DPP-4 inhibitors are safe and do not increase any cardiovascular outcomes. According to the Cardiovascular and Renal Microvascular Outcome Study With Linagliptin (CARMELINA) trial [81], primary outcomes (first occurrence of the composite of CV death, nonfatal myocardial infarction, or nonfatal stroke) occurred in 12.4% of individuals on linagliptin. It provided evidence regarding the CV safety of linagliptin without any clear CV benefits. According to the Trial Evaluating Cardiovascular Outcomes With Sitagliptin (TECOS), sitagliptin had no effect on cardiovascular death, myocardial infarction, stroke, or unstable angina and is safer than saxagliptin or alogliptin [82]. Table 7 details the consensus recommendations.

**Table 7:** Consensus recommendations for management of T2D and CVD. T2D: type 2 diabetes; CVD: cardiovascular disease; SGLT-2: sodium-glucose cotransporter-2; GLP-1: glucagon-like peptide-1; DPP-4: dipeptidyl peptidase-4

Consensus recommendations
A comprehensive strategy that includes lifestyle modifications, lipid management, blood pressure control, and glycemic management should be prioritized.
SGLT-2 inhibitors and GLP-1 receptor agonists with proven cardiovascular benefits should be prioritized in individuals with T2D and established CVD or high CV risk.
Add SGLT-2 inhibitors or GLP-1 receptor agonists along with metformin. If the target HbA1c is not reached, then patients on SGLT-2 inhibitors should be added to GLP-1 receptor agonists or vice versa.
DPP-4 inhibitors are recommended for use in individuals with T2D and CVD due to their neutral cardiovascular profile, offering effective glycemic control without increasing the risk of major adverse cardiovascular events.

Type 2 Diabetes Mellitus and Chronic Kidney Disease 

CKD is estimated to impact 50% of patients with T2D worldwide [83]. A systematic review and meta-analysis reported a pooled CKD prevalence of 27% among individuals with T2D [84]. In India, the Start India Project found that the prevalence of CKD in people with diabetes exceeds 40% [85]. Guidelines recommend lifestyle modifications, glycemic control, CV risk management, and blood pressure regulation using renin-angiotensin-aldosterone system (RAAS) inhibitors, such as ACEIs and ARBs. Metformin, combined with SGLT-2 inhibitors, is advised as the first-line treatment, while GLP-1 receptor agonists are suggested as second-line agents to reduce albuminuria and cardiovascular risk [10,11]. Initiating SGLT-2 inhibitors is not recommended for individuals with an eGFR below 20 mL/min/1.73 m², though the exact threshold may vary depending on the specific agent. However, the medication may be continued in patients who are already on this treatment [86].

Guidelines: Individuals with proteinuria can be managed by ACEIs or ARBs and SGLT-2 inhibitors unless contraindicated or issues with tolerability [9]. A joint consensus from KDIGO and ADA recommends SGLT-2 inhibitors with proven kidney benefits for patients with T2D, CKD, and an eGFR greater than 20 mL/min/1.73 m² [87]. SGLT-2 inhibitors have been shown to reduce albuminuria and the risk of CKD progression, as well as lower mortality and cardiovascular event rates in adults with T2D and CKD [87,88]. GLP-1 receptor agonists are recommended for patients with T2D and CKD who have not achieved glycemic targets despite the use of metformin and SGLT-2 inhibitors or who are unable to use those medications [87].

Evidence for SGLT-2 inhibitors: A systematic review and meta-analysis (13 studies, n=90413) reported that SGLT-2 inhibitors reduced the risk of renal disease progression by 37% (RR: 0.63, 95% CI (0.58-0.69)) and acute kidney injury by 23% (0.77, 0.70-0.84) in individuals with and without T2D [89]. A systematic review and meta-analysis by Zhang et al. [90] (eight studies, n=9367) reported that SGLT-2 inhibitors reduced serum uric acid levels in individuals with CKD. According to the Dapagliflozin in Patients With Chronic Kidney Disease (DAPA-CKD) study [91], in individuals with CKD and with/without T2D, receiving dapagliflozin (10 mg), the primary composite outcome (first occurrence of any of the following: a decline of at least 50% in eGFR, the onset of end-stage kidney disease (ESKD), an eGFR of < 15 mL/min/1.73 m², or death from renal or CV causes) occurred in 9.2% of individuals. The hazard ratio for the sustained decline in the eGFR (at least 50%), ESKD, or death was 0.56 (95% CI, 0.45-0.68), p < 0.001. A prespecified analysis of the DAPA-CKD trial has reported that dapagliflozin significantly reduced albuminuria (reduction in urinary albumin-to-creatinine ratio by 29.3% (95% CI, (-33.1 to -25.2)); p < 0.0001) in individuals with T2D [92]. According to the DIAMOND trial [93], dapagliflozin (10 mg), prescribed for six weeks, when compared to placebo, reduced measured GFR (-6.6 mL/minute per 1.73 m² (-9.0 to -4.2; p < 0.0001)) and body weight in non-diabetic individuals with CKD. According to the Empagliflozin Outcome Trial in Patients With Chronic Heart Failure and a Reduced Ejection Fraction (EMPEROR-Reduced) trial [94] in individuals with/without T2D, empagliflozin (10 mg)-treated individuals had a lower risk of serious renal outcomes and a reduction in eGFR (-0.55 minute per 1.73 m²) when compared to placebo. According to the EMPA-KIDNEY trial [95], in individuals with CKD, the primary outcomes (onset of ESKD, decrease in eGFR (< 10 mL/minute/1.73 m²), decrease in eGFR of at least 40% from baseline, or death from renal causes) occurred in 13% of individuals on empagliflozin.

Evidence for GLP-1 receptor agonists: A systematic review and meta-analysis (eight studies, n=27639) in individuals with T2D and ESRD, by Krisanapan et al. [96], reported that GLP-1 receptor agonists decreased eGFR from −0.6 to −0.1 mL/minute/1.73 m²/month and had a good safety profile. A meta-analysis by Simental-Mendía et al. [97] (18 studies, n=12192), reported that GLP-1 receptor agonists reduced urinary albumin excretion (WMD: −18.01 mg/day, 95% CI (−31.20, −4.82), p=.007) and the albumin-to-creatinine ratio (WMD: −6.74 mg/g, 95% CI (−12.64, −0.85), p=0.03). A meta-analysis (seven studies, n=56004) of the Researching Cardiovascular Events With a Weekly Incretin in Diabetes (REWIND) and Peptide Innovation for Early Diabetes Treatment (PIONEER 6) trials reported that GLP-1 receptor agonists reduced all-cause mortality by 11% and microalbuminuria (HR: 0.76 (0.68-0.86), p=0.003) when compared to placebo in individuals with T2D [98]. Kristensen et al. [99] reported a reduction in renal composite outcomes (new-onset macroalbuminuria, decline in eGFR, ESKD, or death) by 17% in individuals with T2D on GLP-1 receptor agonists.

Evidence for DPP-4 inhibitors: According to the CARMELINA trial [81], primary kidney outcomes (time to the first adjudicated mortality owing to renal failure, ESRD, or a persistent 40% or more drop in eGFR from baseline) were noted in 9.4% of individuals on linagliptin. The progression of albuminuria was less in the linagliptin group when compared to the placebo. According to a study by Karimifar et al. [100], individuals with diabetic nephropathy on linagliptin had a reduced urine albumin-creatinine ratio and a higher percentage of improvement in albuminuria when compared to the control (68.3% vs. 25%, p < 0.001).

Evidence for combination therapy: According to the DELIGHT study [55], in individuals with T2D and CKD, the dapagliflozin (10 mg) and saxagliptin (2.5 mg) combination at the end of 24 weeks reduced the urine albumin creatinine ratio (−38.0% (−48.2 to −25.8), p < 0.0001) when compared to dapagliflozin monotherapy and HbA1c (−0.58% (−0.80 to −0.37), p < 0.0001) when compared to placebo. A systematic review and meta-analysis in individuals with T2D by Ahmad et al. [101] (13 studies, n=1445) reported that SGLT-2 inhibitors + GLP-1 receptor agonists had shown favorable kidney outcomes (reduced urine albumin creatinine ratio and macroalbuminuria). Table 8 details the consensus recommendations.

**Table 8:** Consensus recommendations for management of T2D and CKD. T2D: type 2 diabetes; CKD: chronic kidney disease; SGLT-2: sodium-glucose cotransporter-2; GLP-1: glucagon-like peptide-1; UACR: urine albumin-creatinine ratio; HbA1c: glycated hemoglobin; eGFR: estimated glomerular filtration rate

Consensus recommendations
Regular monitoring of renal function (eGFR) is crucial, and adjustments to medication should be made based on renal status.
SGLT-2 inhibitors can be used in individuals with eGFR > 20 mL/min/1.73m^2 ^(dapagliflozin (25 mL/min/1.73m^2^, empagliflozin (20 mL/min/1.73m^2^)) and a UACR ≥ 200 mg/g.
Add SGLT-2 inhibitors or GLP-1 receptor agonists along with metformin. If the target HbA1c is not reached, then patients on SGLT-2 inhibitors should be added to GLP-1 receptor agonists or vice versa.

Type 2 Diabetes, Obesity, and Dyslipidemia

Obesity is a major risk factor for T2D and contributes to insulin resistance, while the presence of T2D can, in turn, increase the risk of weight gain due to the intake of excess calories to meet the increased energy demands caused by insulin resistance [102]. According to a systematic review and meta-analysis, the pooled prevalence of overweight and obesity in individuals with T2D was 35.6% and 25.6%, respectively [102]. Pharmacotherapy for weight loss should be considered when lifestyle interventions alone fail to achieve the desired weight loss goals. The ADA recommends a 5% weight reduction for individuals with T2D who are overweight or obese [10]. Guidelines recommend the use of GLP-1 receptor agonists and SGLT-2 inhibitors for individuals with T2D and obesity [9-11].

T2D and dyslipidemia are independent risk factors for CVD, and their coexistence significantly heightens the risk of CVD in individuals with T2D. Globally, a high prevalence of dyslipidemia has been observed among people with T2D, with studies reporting rates exceeding 65% [103-106]. ADA recommends statins for T2D individuals with dyslipidemia and additional CV risk factors [59]. AACE recommends the use of high- and moderate-intensity statins, fibrates, and ezetimibe for the treatment of dyslipidemia [42].

One of the most promising advancements in diabetes treatment is the emergence of dual glucose-dependent insulinotropic polypeptide (GIP) and GLP-1 receptor agonists, commonly referred to as “twincretins.” These agents represent a novel therapeutic approach for managing T2D and obesity. Findings from the SURPASS clinical trials demonstrate that tirzepatide significantly improved glycemic control (HbA1c reduction ranging from 1.91% to 2.11%) and promoted substantial weight loss (7 to 9.5 kg) [107]. While these agents are not yet widely accessible in routine Indian clinical practice and were not the primary focus of the consensus, we acknowledge their future role and have mentioned them as a potential extension of simplified glycemic care strategies.

Guidelines: GLP-1 receptor agonists and SGLT-2 inhibitors are advised in individuals with overweight or obesity [11,42]. DPP-4 inhibitors are used as an alternative or add-on therapy [42].

Evidence for SGLT-2 inhibitors: A systematic review and meta-analysis by Wang et al. [108] (18 studies, n=1063) in individuals with T2D reported that SGLT-2 inhibitors reduced visceral adipose tissue (SMD=−1.42, 95% CI (−2.02, −0.82), p < 0.0001), body weight (MD=−2.60, 95% CI (−3.30, −1.89), p < 0.0001), waist circumference (MD=−3.65, 95% CI (−4.10, −3.21), p < 0.0001), and BMI (MD=−0.81, 95% CI (−0.91, −0.71), p < 0.0001). Li et al. [109] reported that SGLT-2 inhibitors are not associated with an increased risk of dyslipidemia, and they significantly decrease triglycerides (-0.12 mmol/l). A systematic review by Sánchez-García et al. [110] and a meta-analysis by Bechmann et al. [111] reported that SGLT-2 inhibitors increased low-density lipoprotein cholesterol (LDL-C) and high-density lipoprotein cholesterol (HDL-C). A significant reduction is seen in triglyceride levels.

Evidence for GLP-1 receptor agonists: A systematic review and meta-analysis by Li et al. [112] (29 studies, n=10333) reported that GLP-1 receptor agonists reduced weight (−14.13 kg, 95% CI (−16.49, −11.73)) and HbA1c (−0.33%, 95% CI (−0.41, −0.25)). A systematic review by Anam et al. [113] (12 studies) found that semaglutide is safe and effective for weight loss in individuals with T2D and obesity. A systematic review and meta-analysis by Yao et al. [114] (76 studies, n=39,256) found that CagriSema (semaglutide with cagrilintide) led to significant weight loss (−14.03 kg, 95% CI (−17.05 to −11.00)), followed by tirzepatide (−8.47 kg, 95% CI (−9.68 to −7.26)). Additionally, semaglutide was shown to reduce LDL-C (−0.16 mmol/L, 95% CI (−0.30 to −0.02)) and total cholesterol (−0.48 mmol/L, 95% CI (−0.84 to −0.11)). A systematic review and meta-analysis in overweight/obese patients with/without diabetes mellitus reported that GLP-1 receptor agonists were superior to SGLT-2 inhibitors for HbA1c (MD: -0.39%, 95% CI (-0.70 to -0.08)) and weight reduction (MD: -11.51 kg, 95% CI (-12.83 to -10.21)) [115]. The PIONEER studies (1-10) evaluated semaglutide in comparison with other GLP-1 receptor agonists and oral antihyperglycemic drugs to assess weight changes and cardiovascular safety. All these studies consistently reported weight loss in individuals treated with semaglutide [116]. A systematic review and meta-analysis by Chae et al. [117] (26 studies, n=22290) reported that GLP-1 receptor agonists reduced total cholesterol (-5.20% p; -6.39% p) and LDL-C (-4.32% p; -8.17% p). Table 9 details the consensus recommendations.

**Table 9:** Consensus recommendations for management of T2D, obesity, and dyslipidemia T2D: type 2 diabetes; SGLT-2: sodium-glucose cotransporter-2; GLP-1: glucagon-like peptide-1; DPP-4: dipeptidyl peptidase-4

Consensus recommendations
GLP-1 receptor agonists and SGLT-2 inhibitors are recommended for their dual effects on weight loss and glycemic control.
DPP-4 inhibitors are weight-neutral, and metformin causes modest weight reduction.
Statins should be prescribed for all T2D patients with dyslipidemia, especially those with additional cardiovascular risk factors.

**Figure 3:**
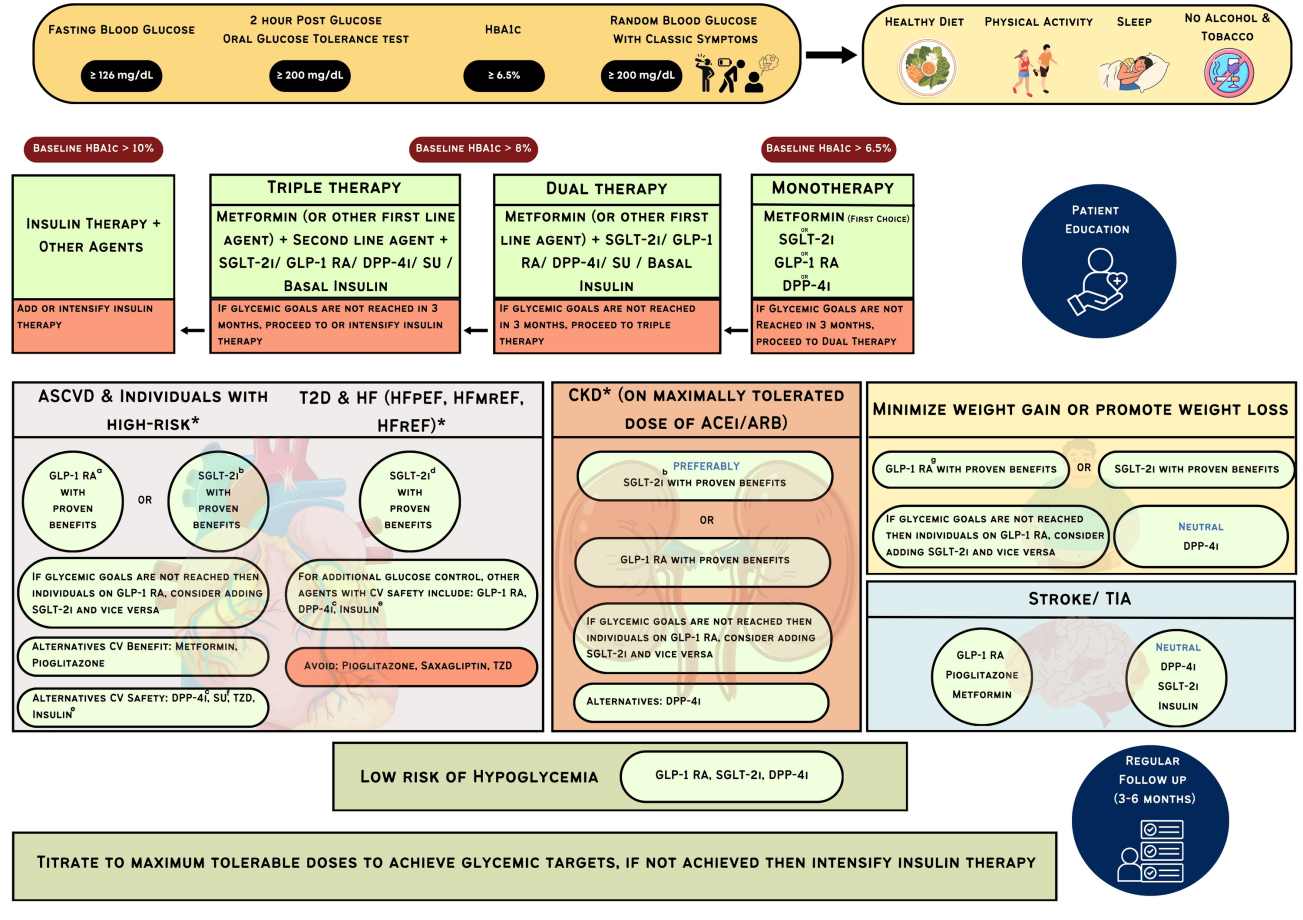
Management of T2D and comorbidities (lifestyle modifications and antihyperglycemic drugs). ACEi: angiotensin-converting enzyme inhibitor; ARB: angiotensin receptor blocker; ASCVD: atherosclerotic cardiovascular disease; CKD: chronic kidney disease; CVD: cardiovascular disease; DPP-4i: dipeptidyl peptidase 4 inhibitor; GLP-1 RA: glucagon-like peptide 1 receptor agonist; HF: heart failure; HFpEF: heart failure with preserved ejection fraction; HFrEF: heart failure with reduced ejection fraction; HFmEF: heart failure with mid-range ejection fraction; SGLT2i: sodium-glucose cotransporter 2 inhibitor; T2D: type 2 diabetes; TZD: thiazolidinedione; SU: sulphonylureas * In individuals with HF, CKD, established CVD, or multiple risk factors for CVD, the decision to use GLP-1 RA or SGLT-2i with proven benefit should be independent of background use of metformin. TZD: Low dose is better tolerated. ^a^ GLP-1RA with proven CV benefit: liraglutide, semaglutide, exenatide; ^b^ SGLT-2 inhibitors with proven CV and renal benefit: empagliflozin, canagliflozin, dapagliflozin; ^c^ DPP-4 inhibitors: should not be used in patients on GLP-1 RAs; ^d^ SGLT-2 inhibitors for heart failure: empagliflozin, dapagliflozin; ^e^ Insulin glargine or degludec; ^f^ SU: newer sulphonylureas are preferred; ^g^ GLP-1 RA for weight loss: semaglutide, liraglutide, dulaglutide, exenatide. Modified and adapted from: Samson et al. [42] and Davies et al. [11] Image credit: Dr. Dinesh Jiwane

Figure 3 summarizes the comprehensive management of T2D and comorbidities.

## Conclusions

The rising prevalence of T2D in India, coupled with its association with various macrovascular and microvascular complications, presents a significant public health challenge. This consensus on "Simplified Glycemic Care in India" is an effort to address the complexities of T2D management, aiming to develop a practical, patient-centered approach that enhances treatment adherence, reduces the pill burden, and improves clinical outcomes. Given the high costs and psychological burden associated with T2D, the importance of a streamlined treatment regimen is the need of the hour. The expert panel's recommendations highlight the pivotal role of combination therapy, particularly the use of SGLT-2 inhibitors and DPP-4 inhibitors, either alone or alongside metformin, to provide effective glycemic control while offering additional cardiovascular and renal benefits. These recommendations emphasize the need for individualized treatment plans, considering factors such as patient comorbidities, tolerability, and cost-effectiveness, to achieve optimal outcomes in glycemic control. In summary, the consensus provides actionable strategies and evidence-based recommendations for the management of T2D, reinforcing the importance of a simplified, patient-centered approach to glycemic care.
